# Structure-based virtual screening of essential Mycobacterium tuberculosis enzymes AspS and KatG for potential inhibitors

**DOI:** 10.6026/97320630017101

**Published:** 2021-01-31

**Authors:** Andrew P Collins, Waleed Abdelfattah

**Affiliations:** 1 University of Central Florida College of Medicine, Orlando, Florida; 2 University of Washington, Seattle, USA

**Keywords:** Structure, virtual screening, AspS, KatG

## Abstract

Mycobacterium tuberculosis (TB) is a leading global cause of disease-related death. Recent works have studied metabolic pathways of the mycobacterium, highlighting essential enzymes to target via competitive inhibition through computational molecular modeling to suppress the organism's life cycle. We used the Protein Databank (PDB), the UniProt Knowledgebase and the iDock server in this study. In vitro toxicity screening and pharmacokinetic properties were assessed to determine potential ligand safety and drug properties. Our results have revealed five and nine potential ligands for the enzymes AspS and KatG respectively. The KatG active site has displayed binding affinities of -13.443 to -12.895 kcal/mol, while AspS ligands range from -6.580 to -6.490kcal/mol. The intermolecular forces responsible for the differing binding affinities of each enzyme are primarily Coulombic interactions for AspS, versus Coulombic and extensive hydrogen bonding interactions in KatG.

## Background:

Mycobacterium tuberculosis is estimated to infect nearly one quarter of the world's population, roughly 1.7 billion people. Although the disease is latent in most cases, over 10 million patients are infected with the active disease each year, with an overall mortality rate as high as 12.3% [1,2]. The prevalence of varying degrees of drug-resistant strains is increasing, making disease containment a clinical challenge [3]. Many of those infected today do not have access to the antibiotic protocols or are infected with multi-drug resistant strains that are not manageable through current pharmaceuticals. In 2016, the disease ranked amongst the top 10 global causes of death [4]. For decades, the discovery of drugs depended on trial and error techniques to sort through molecular libraries and test compound properties. Advances in computer-aided drug design have led to the creation of virtual molecule libraries with millions of compounds. As research is conducted to analyze the metabolic pathways of specific disease-causing organisms, these drug libraries can be utilized to fit molecules to the organism's active site in order to inhibit enzymatic function. This process of virtual screening and structure-based drug discovery has become an essential method to rapidly develop cost-efficient drug molecules to fit known enzyme active sites [5]. We have fulfilled a review of previous literature outlining enzymatic processes of the mycobacterium, identifying those essential to the survival of the organism. These enzymes are fit with small, non-toxic-organic molecules to block the enzymatic function by working as competitive inhibitors. Since these ligands are introduced as novel molecules to the body, they must undergo screening for absorption, distribution, metabolism, and excretion (ADME) related physical properties and in silico toxicity models in order to avoid acute toxicity. The goal of TB drug development is to shorten the lifespan of the active mycobacterium and decrease the detrimental symptoms caused by the disease as well.

## Methods:

### Modeling Rationale:

Docking of novel ligands to the AspS and KatG enzymes were conducted through the online high-throughput virtual screening tool iDock. The iDock server docks ligands based on several structural components, such as the ligand's size, molecular weight, rotatable bonds, hydrogen (H)- bond acceptor locations, etc. in order to rank novel ligands based on their affinity for the enzyme's active site. Overall, enzymes will have docking parameters assessed with nearly 1M ligands. The output from this molecular screening method will display ligand-binding affinities, and the highest 2,500 scoring potential molecules were then further screened. These iDock-screened molecules were then processed through a toxicity screen using Swiss ADME, which predicted the absorption, distribution, metabolism, excretion, and toxicity of the ligands. This web tool has yielded accurate predictions for these properties in similar ligand screening studies [6]. Through these two screening methods, each enzyme's list of approximately 1M potential ligands was narrowed down to five and nine potential ligands for AspS and KatG, respectively.

### Determining Essential Target Enzymes:

**Figure 1:**
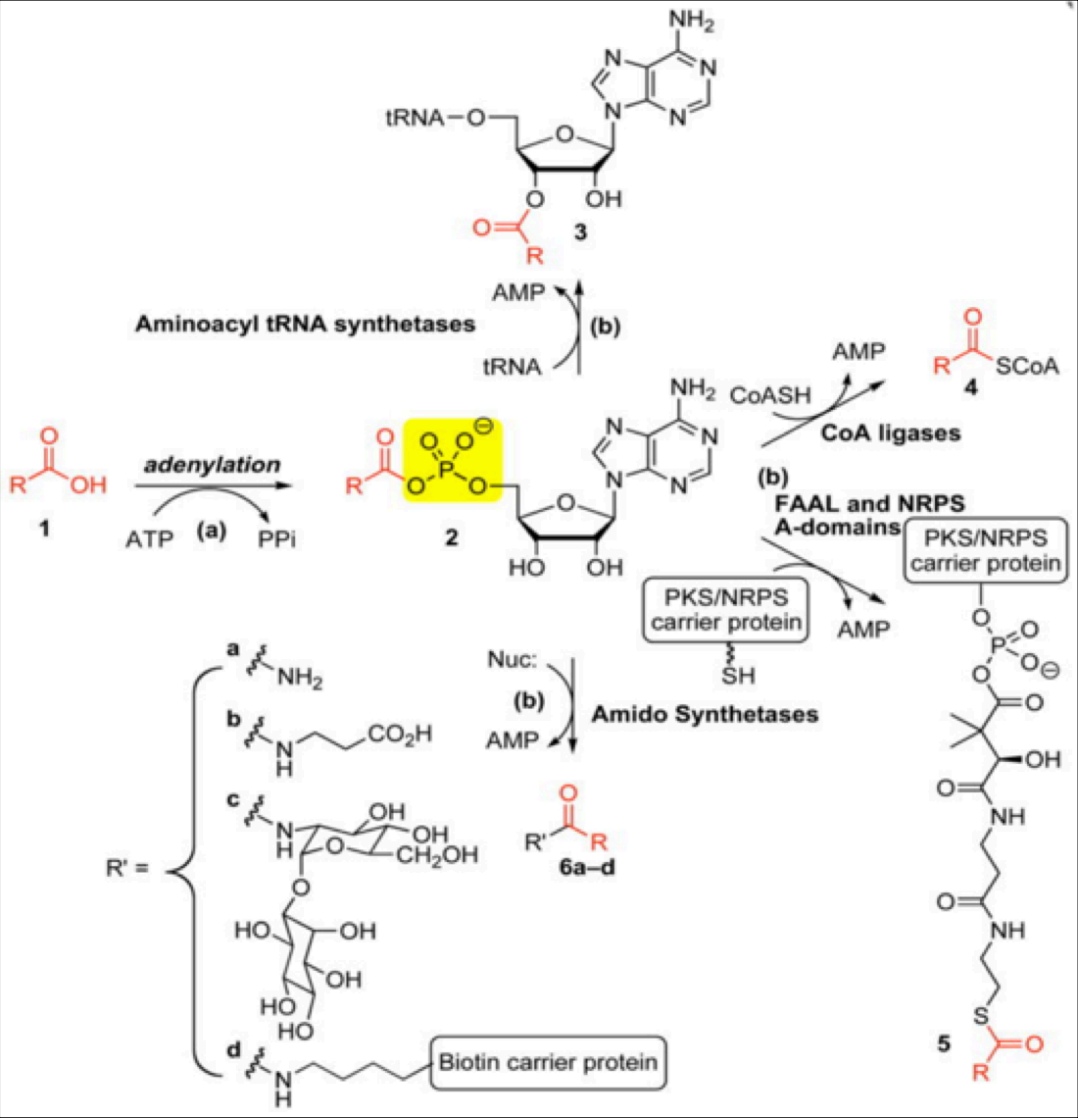
Overview of adenylating enzyme mechanism [7]

Determining specific enzymes for virtual screening required an analysis of metabolic pathways of Mycobacterium tuberculosis to identify enzymes essential to the survival of the organism. The pathway targeted in this study involves adenylating enzymes, specifically those that fulfill aminoacyl tRNA synthetase (AARS) ribosomal protein biosynthesis. The proteins involved in this pathway activate ribosomal subunits or load amino acids into the tRNA molecule. The specific enzymes AspS and KatG play essential adenylating roles in ribosomal protein biosynthesis, and without either of these proteins functioning properly, the cell cannot translate proteins sufficiently and the propagation of the mycobacterium will decrease greatly. Recent studies have explored the adenylation pathway and mechanism of several TB enzymes [7]. They concluded that many of the proteins involved in the pathway are essential to the ribosomal protein biosynthesis and overall translational functions of the bacterium. The proteins AspS and KatG were chosen from a group of enzymes discussed in the group's research, due to their well-known 3-dimensional structure, and other physical features such as active site size, accessibility, and affinity for non-toxic novel ligands. Adenylating enzymes catalyze a two-step reaction shown in Figure 1. The first reaction involves the binding of a carboxylic acid substrate (often an amino acid) and ATP, forming an acyl-adenylate. The second reaction occurs as the enzyme binds an acceptor molecule and transfers the acyl-adenylate to a nucleophilic molecule. The specific enzymes targeted, which play a role in AARS, follow a similar mechanism to that shown in Figure 1. By creating a molecule mimicking the acyl-adenylating intermediate, the enzymes responsible for this essential reaction will become competitively inhibited, decreasing enzymatic function [7,8].

### Preparation for Virtual Screening:

In order to use these enzymes as targets, their crystal structures must be sequenced and available. We chose to use the Protein Databank (PDB) to search for 3D crystal structures of the essential enzymes, only two of which had the structures known and available. This process narrowed the list of possible enzymes to target to only two: KatG and AspS. To sequence the enzyme, the UniProt Knowledgebase was utilized. The database determined the full enzyme sequence as well as specific residues comprising the active site. These residues responsible for active site binding and transition state stabilization yielded locations on the enzyme to target through virtual screening. To visualize and create a file for the virtual screening tool iDock to analyze, the University of California San Francisco's molecular modeling program Chimera was utilized. This program was utilized for manipulation of the structure to show or hide certain parameters of the enzyme, including specific side chains, available H-bonds, and ionic interactions.

### Enzyme Virtual Screening:

**Figure 2:**
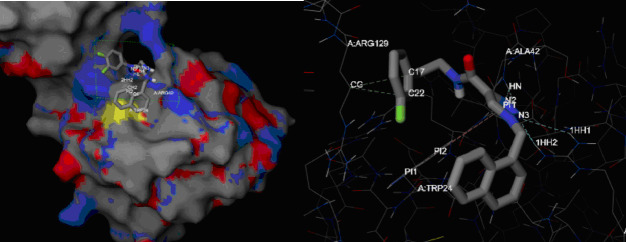
iDock output of a potential ligand interacting with the AspS active site.

The virtual screening program iDock was utilized to assess ligand interactions with the essential enzymes, AspS and KatG. The server has a database containing approximately 24M ligands that the enzyme can undergo screening with. The active site was used as a box model, in which the virtual screening tool, iDock, sorted through the database's molecular structure to analyze the ligand affinity for enzyme's active sites. The iDock server utilizes a machine-learning scoring function, allowing it to improve on its scoring algorithm accuracy, due to its ability to recognize proper molecular positioning in 3-D space [6]. These machine-learning algorithms have the ability to yield very accurate results and tend to outperform classical scoring functions at binding affinity predictions for complex enzyme-ligand interactions [6]. The specific physical and structural ligand parameters set for iDock were chosen to mimic common organic molecules that are likely permeable to the gastrointestinal tract (thus readily absorbed into the blood stream via oral or suppository administration). The specified setting included: (1) molecular weight between 350-551 g/mol; (2) presence of 1-5 H-bond donors; (3) presence of 1-7 H-bond acceptors; (4) apolar desolvation of 0-6 kcal/mol; (5) polar desolvation of -50-0 kcal/mol; (6) partition coefficient xlogP of 1-5; (7) polar surface area tPSA between 50-140 angstroms2; (8) net charge of zero; and (9) containing 3-8 rotatable bonds. The specific settings chosen limited the potential number of ligands from the database's 24M to 978,098 unique compounds. Figure 2 depicts an output from the iDock server, showing the charged side chain groups highlighted in green, and the polar regions of the molecule indicated in blue, displaying physical interactions between the enzyme-ligand complex. High iDock scores correlate with high ligand-enzyme affinity, while low scores indicate low enzyme-ligand affinity. Those with high iDock scores typically show a more visibly compact enzyme-ligand complex than those with lower scores, which is expected, due to their differences in affinity. These 3-D images were utilized to analyze the spatial parameters of ionic interactions, dipole-dipole interactions, and also H-bonding interactions of the enzyme-ligand complexes. Sorting the ligand results by iDock score, the 2,500 top scoring ligands underwent further screening for pharmacokinetic properties.

### Pharmacokinetic Analysis of Ligands:

From the generated iDock results for each enzyme, the top 2,500 scoring ligands were assessed for pharmacokinetic properties, drug-like nature, and medicinal chemistry friendliness through the online Swiss ADME server. The server computes projected compound interactions with the human body related to absorption, distribution, metabolism, and excretion, such as gastrointestinal (GI) absorption, blood-brain barrier (BBB) permeability, various cytochrome (Cyt) C inhibition, bioavailability score, synthetic accessibility, and many others [9]. The Swiss ADME server narrowed the list of 2,500 high-affinity ligands per enzyme to our resulting five and nine possible ligands, based on the projected interactions they have with the human body. Through the results from this server, ligand processing was completed based on five separate properties: (1) high GI tract absorption; (2) low blood-brain barrier permeability; (3) lack of specific cytochrome inhibition (for CYP1A2, CYP2C19, CYP2C9, CYP2D6, and CYP3A4); (4) medium-high bioavailability scores; and (5) high synthetic accessibility. Ligands that fulfill these criteria while still maintaining high iDock scores took precedence as potential ligands.

## Results:

The AspS binding site contains four crucial residues that participate in Coulombic interactions with ligand molecules. These are found as four aspartate residues at the 170, 216, 448, and 489 positions. The ligand molecules from the iDock database yielded scoring results from the server (iDock score), representing enzyme-binding affinity for the ligand. The results in Table 1 (see PDF) list these potential ligands after iDock affinity screening and Swiss ADME toxicity analysis. International Union of Pure and Applied Chemistry (IUPAC) molecule names are listed for identification as well. The five molecules successfully screened for the AspS active site ranged in binding affinity from -6.580 to -6.490 kcal/mol. The active site and ligands interacted primarily through Coulombic interactions. The AspS ADME properties are depicted in Table 1 (see PDF). These results indicate that all of these potential ligands have high gastrointestinal absorption levels and low blood brain barrier permeability. Additionally, none of these ligands inhibit the functions of the various screened cytochrome P450 enzymes. The synthetic accessibility scores are graded on a 0-10 scale, with 0 equating to very accessible and 10 not accessible, based on ADME properties. Since all of these values lie between 2 and 3, the ligands have similarly high synthetic accessibility scores (1 = very easy access, 10 = very difficult access). Thus, these five ligands passed the ADME screening criteria and are possible effective inhibitors of AspS. These molecules screened for AspS ranged in molecular weight from 374.43 to 352.39 g/mol. The KatG active site contains three residues that participate in ligand binding at positions 107, 108, 270, and 321; these interacting residues are tryptophan, histidine, histidine, and tryptophan, respectively. The results in Table 2 (see PDF) list these ligands after a screening through iDock for binding affinity and Swiss ADME for toxicity analysis, with IUPAC chemical formulas. The nine molecules successfully screened for the AspS active site displayed very high binding affinity, ranging from -13.443 to -12.895 kcal/mol. This strong binding affinity is likely due to the many H-bonding interactions in addition to the Coulombic ion interactions as well. Table 2 (see PDF) shows the Swiss ADME results for KatG. Similar to the AspS potential enzymes, each of these was screened for the same properties and has strong GI absorption, and low BBB permeability. Synthetic accessibility ranged from 2.42 to 4.53, indicating high to moderate bioavailability of the ligands. These molecules screened for KatG ranged in molecular weight from 442.39 to 351.33 g/mol.

## Discussion:

The aminoacyl tRNA synthetase ribosomal protein biosynthesis serves as an essential enzyme in the metabolic function and life cycle of Mycobacterium tuberculosis. In recent years, the AARS pathway has emerged as a potential target for the discovery of novel inhibiting ligands against the mycobacterium [7,8]. In this study, we have focused efforts on identifying small-organic molecule inhibitors against the AARS pathway, which could serve as lead molecules for further development of novel ligands used against the TB disease process. Modern-day drug discovery often utilizes high-throughput structure-based, target-based, and phenotypic screening, all of which can provide high output for molecular discovery, but often come at a high cost to researchers. In contrast to these methods, molecular virtual screening is inexpensive and can efficiently screen enzyme-ligand complexes in silico to reduce the number of in vitro ligands for testing [5]. For this reason, virtual screening has gained a crucial foothold in the early processes of novel drug-discovery. Although Mycobacterium tuberculosis enzymes have been a target of virtual screening for several studies, none have analyzed the protein adenylation pathway yet. Our findings have identified several potential ligands used for competitive inhibition of the AspS and KatG essential enzymes of the mycobacterium. While the KatG enzymatic site was shown to be a deep funnel-shape containing several H-bonding interactions for ligands, the AspS active site was more surface level and utilized mostly Coulombic interactions for ligand binding. This was noted in the ligand binding affinities of each enzyme; KatG had a very high average ligand-enzyme affinity, while AspS had an average affinity of roughly half of the strength. These enzymes were analyzed in silico for predicted human toxicity, as well as ADME pharmacokinetic properties. Although they have not yet been tested in vitro, the computational models used in this study have shown them to be promising ligands against the AARS pathway of Mycobacterium tuberculosis.

## Conclusion:

With the high prevalence of Mycobacterium tuberculosis in Eastern nations and developing countries, the market needs new cures that can combat the emerging drug-resistant strains. Experimental and computational tools are useful options that are used for novel drug discovery [5]. The low cost and quick results found through virtual screening of ligand molecules for specific enzymes makes these computational methods essential for speeding up pharmaceutical exploration [5]. With the discovery of several adenylating metabolic pathways, new enzymatic targets are identified for ligand docking to competitively inhibit enzyme function. From the iDock server's 24M compound database, approximately 1M of these were screened against the essential adenylating enzymes, AspS and KatG. From these initial results displaying enzyme-ligand binding affinity, the ligands were screened for pharmaceutical properties,such as drug likeness, bioavailability, and cytochrome inhibition as well as absorption, distribution, metabolism, and excretion patterns. Through this screening process, five and nine promising ligands were found for the enzymes AspS and KatG, respectively. This work is proposed to assist in future clinical trials that seek novel inhibitory compounds to combat TB, leading to accelerated drug discovery.

## Author contributions:

All authors contributed to data analysis, drafting, or revision of the article, gave final approval of the final version to be published, and agreed to be accountable for all aspects of the work.
